# Prognostic value of rising mean platelet volume during hospitalization in patients with ST-segment elevation myocardial infarction treated with primary percutaneous coronary intervention

**DOI:** 10.1186/s12872-018-0970-6

**Published:** 2018-12-07

**Authors:** Eyup Avci, Tuncay Kiris, Aykan Çelik, Eser Variş, Fatma Kayaalti Esin, Diyar Köprülü, Hasan Kadi

**Affiliations:** 1 Department of Cardiology, Medical School, Balikesir University, Balikesir, Turkey; 2 Department of Cardiology, Ataturk Training and Research Hospital, Izmir Katip Celebi University, Basın Sitesi, 35360 Izmir, Turkey; 3 Department of Cardiology, Dr. Burhan Nalbantoğlu State Hospital, Nicosia, Cyprus; 4 Department of Cardiology, Ordu State Hospital, Ordu, Turkey

**Keywords:** Mean platelet volume, ST segment elevation myocardial infarction, Mortality

## Abstract

**Background:**

The prognostic significance of changes in mean platelet volume (MPV) during hospitalization in ST segment elevation myocardial infarction (STEMI) patients underwent primary percutaneous coronary intervention (pPCI) has not been previously evaluated. The aim of this study was to determine the association of in-hospital changes in MPV and mortality in these patients.

**Methods:**

Four hundred eighty consecutive STEMI patients were enrolled in this retrospective study. The patients were grouped as survivors (*n* = 370) or non-survivors (*n* = 110). MPV at admission, and at 48–72 h was evaluated. Change in MPV (MPV at 48–72 h minus MPV on admission) was defined as ΔMPV.

**Results:**

At follow-up, long-term mortality was 23%. The non-survivors had a high ΔMPV than survivors (0.37 (− 0.1–0.89) vs 0.79 (0.30–1.40) fL, *p* <  0.001). A high ΔMPV was an independent predictor of all cause mortality ((HR: 1.301 [1.070–1.582], *p* = 0.008). Morever, for long-term mortality, the AUC of a multivariable model that included age, LVEF, Killip class, and history of stroke/TIA was 0.781 (95% CI:0.731–0.832, *p* <  0.001). When ΔMPV was added to a multivariable model, the AUC was 0.800 (95% CI: 0.750–0.848, z = 2.256, difference *p* = 0.0241, Fig. 1). Also, the addition of ΔMPV to a multivariable model was associated with a significant net reclassification improvement estimated at 24.5% (*p* = 0.027) and an integrated discrimination improvement of 0.014 (*p* = 0.0198).

**Conclusions:**

Rising MPV during hospitalization in STEMI patients treated with pPCI was associated with long-term mortality.

## Introduction

Platelets (PLT) play an important role in in both initiation and progression of acute coronary syndromes (ACS) [1, 2]. Previous studies have shown that platelets had pro-inflammatory activity independently of their actions of haemostasis and vascular thrombosis [3–5]. Mean platelet volume (MPV) is an indicator of platelet activation [6]. Large PLTs are more active and have higher thrombotic potential compared with small PLTs. They are also more intense, and they have higher thromboxane A2 levels, and more glycoprotein Ib and IIb/IIIa receptors. Thus enabling PLT to aggregate more quickly with collagen more than the small PLTs [7].

In the recent years, numerous studies have been shown that the increased MPV at admission was associated with long-term mortality in patients with ACS [8–10]. Morever, an in-hospital increase in MPV after admission was found to be predictor of mortality in both non-ST segment elevation myocardial infarction and critical patients [11, 12]. There is no data about the association of in-hospital MPV change with mortality in patients with ST-elevation myocardial infarction (STEMI) treated with primary percutaneous coronary intervention (PCI).

The aim of the present study was to investigate the relationship of an in-hospital increase in MPV with long term mortality in STEM patient underwent primary PCI.

## Methods

### Study population

We enrolled 514 STEMI patients who were undergoing primary PCI between January 2008 and June 2015. The patients with malignancy or infectious disease or autoimmune disease or hematologic disease and patients with incompleted data were excluded from this study. The final analysis included 480 patients. Permission of study was obtained by a local ethics committee. STEMI diagnosis was established as typical angina pain lasting > 30 min, with increase in levels of cardiac enzymes (troponin I levels) and electrocardiographic evidence of elevation of the ST segment of > 1 mm in two or more consecutive leads or the presence of new left bundle branch block (LBBB) [13]. We defined hypertension (HT) as the previous use of antihypertensive medication, systolic pressure > 140 mmHg or diastolic pressure > 90 mmHg and Diabetes mellitus as the use of insulin or antidiabetic agents or a fasting glucose level > 126 mg/dL. Hypercholesterolemia was diagnosed as total cholesterol of ≥200 mg/dL. Smoking was defined as a current smoker or not. This study complied with the Declaration of Helsinki.

We defined total mortality as death due to any cause at follow-up and considered as the primary end point. Morever, repeat revascularization, heart failure admission, and stroke/transient ischemic attack (TIA), and 30-day mortality were also evaluated. We obtained follow-up data from the hospital records, patients, and their relatives.

### Procedures

All patients were treated according to the current guidelines.13 Primary PCI was performed using standard techniques via the transfemoral approach by 2-experienced interventional cardiologists. The treatment strategies for each patient were left to the discretion of interventional cardiologists. We obtained angiographic data from the cardiac catheterization laboratory records. The infarct-related artery (IRA) was evaluated based on the thrombolysis in myocardial infarction (TIMI) classification. We defined invasive success in acute phase as reduction to < %20 in IRA obstruction and stenosis with TIMI-3 flow immediately after primary PCI. After angioplasty, all patients were transferred to intensive care unit. Dual antiplatelet therapy, beta-blockers, angiotensin-converting enzyme inhibitors (ACE-I), angiotensin receptor-blocker (ARB), and statins were administered according to current guidelines [13].

The left ventricle ejection fraction (LVEF) was calculated after measuring the end-diastolic and end-systolic left ventricle (LV) volumes in the apical four-chamber and two-chamber views using the modified Simpson’s method.

### Blood sampling and hematological and biochemical analyses

Peripheral blood was obtained for MPV analysis at admission prior to administration of antiplatelet drugs and 48–72 h after admission. Blood samples were collected into standardized tubes containing dipotassium ethylenediaminetetraacetate powder as anticoagulant and stored at room temperature. All measurements were analyzed within 1 h after collection. Change in MPV was defined as ΔMPV (MPV at 48–72 h minus MPV on admission). An extra blood was collected on admission for biochemical analysis. They were evaluated by standard methods.

### Statistical analysis

Statistical analysis was made using SPSS software (version 16.0; SPSS Inc., Chicago, IL, USA). Variables with normal distribution were analyzed using Kolmogorov-Smirnov test and presented as mean ± standart deviation, while those without normal distribution were presented as medians with a range. Categorical variables were presented as number and percentage. The comparisons between groups was carried out using the chi-square test for categorical variables and Student t tests or Mann-Whitney U test for continuous variables. A multivariate cox regression analysis was carried out to evaluate whether ΔMPV was an independent predictor of mortality. Factors with a *p* value of < 0.1 by univariate analysis were included in multivariate cox regression analysis. The predictive values of a multivariable model and a combination of ΔMPV with a multivariable model were estimated by comparing the areas under the receivers operating characteristic (ROC) curve. DeLong’s test was used to compare the AUC from each of models [14], which were analysed by use of NCSS 12 software programme. Morever, the increased discriminative value after the addition of ΔMPV to a multivariable model was also estimated using the Net Reclassification Improvement (NRI) and Integrated Discrimination Improvement [15]. Differences were considered significant at the 2-sided *p* <  0.05 level.

## Results

### Baseline characteristics

Baseline demographic, and clinical characteristics are shown in Table 1. Median follow-up time was 65.9 (41.9–80.4) months. The non-survivors were significantly older (68 ± 13 vs 58 ± 11, *p* <  0.001). The histories of diabetes mellitus, HT, stroke/TIA were more common in non-survivors compared with survivors (Table 1). The frequency of Killip class ≥2 and multi-vessel disease were higher in non-survivors than survivors. Compared with survivors there was a higher proportion of women in non-survivors. The rates of usage of ACE-I/ARB and beta-blockers after discharge were lower in the non-survivors than survivors.

**Table 1:** Baseline characteristics of the study population

Variable	Survivors (*n* = 370)	Non-survivors (*n* = 110)	*P*-value
Age (year)	58 ± 11	68 ± 13	< 0.001
Female *n* (%)	80 (22)	37 (34)	0.010
Hypertension *n* (%)	151 (41)	63 (57)	0.002
Diabetes mellitus *n* (%)	78 (21)	37 (34)	0.007
Hyperlipidemia *n* (%)	76 (21)	24 (22)	0.772
Current smoking *n* (%)	168 (45)	40 (36)	0.098
Previous CAD *n* (%)	64 (17)	27 (25)	0.089
Prior stroke/TIA *n* (%)	5 (1)	9 (8)	< 0.001
Killip class ≥2 *n* (%)	21 (6)	20 (18)	< 0.001
Multi-vessel disease *n* (%)	132 (36)	57 (52)	0.002
GP IIb/IIIa inhibitors *n* (%)	107 (29)	37 (34)	0.343
Medication at discharge			
Beta-blocker *n* (%)	319 (86)	82 (73)	0.004
Statin *n* (%)	312 (84)	85 (77)	0.086
ACE-I/ARB *n* (%)	315 (85)	76 (69)	< 0.001
DAPT *n* (%)	365 (99)	107 (97)	0.322
Infarct related artery			0.097
LAD *n* (%)	170 (46)	50 (46)	
Cx *n* (%)	57 (15)	8 (7)	
RCA *n* (%)	129 (35)	49 (45)	
Others *n* (%)	14 (4)	3 (2)	
Outcomes			
30-day death *n* (%)	0 (0)	19 (17)	< 0.001
Stroke *n* (%)	7 (2)	5 (5)	0.118
HF admission *n* (%)	7 (2)	15 (14)	< 0.001
Myocardial reinfarction *n* (%)	30 (8)	8 (7)	0.776
TVR *n* (%)	45 (12)	11 (10)	0.535

*HF* heart failure, *CAD* coronary artery disease, *TIA* transient ischemic attack, *ACE-I* angiotensin-converting enzyme inhibitors, *ARB* angiotensin receptor blocker, TVR; target vessel revascularization, *DAPT* dual antiplatelet therapy

### Laboratory parameters

Laboratory variables are provided in Table 2. Serum creatinine level at admission was higher in the non-survivors. Compared with survivors, admission hemoglobin level was lower in the non-survivors. There was no significant difference between groups in terms of platelet counts both at admission and at 48–72 h. MPV at 48–72 h was higher in non-survivors than survivors. Compared with survivors, non-survivors had a high ΔMPV value [0.79 (0.30–1.40) vs 0.37 (− 0.1–0.89), *p* < 0.001]. Baseline MPV was similar between groups.

**Table 2:** The laboratory findings of study population

Variable	Survivor (*n* = 370)	Non-survivor (*n* = 110)	*P* value
Total cholesterol (mg/dl)	181 ± 43	163 ± 39	0.001
SCr^a^_adm_ (mg/dl)	0.86 (0.76–1.02)	0.95 (0.80–1.26)	0.048
Hemoglobin (g/dl)	14.0 ± 1.2	13.2 ± 2.2	< 0.001
WBC count (10^3^/mm^3^)	12 ± 4	12 ± 5	0.781
Platelets _adm_(10^3^/mm^3^)	273 ± 78	271 ± 93	0.846
Platelets _48-72h_ (10^3^/mm^3^)	241 ± 73	235 ± 90	0.448
MPV _adm_ ((fL)	9.0 ± 1.6	9.0 ± 1.3	0.648
MPV_48-72h_ (fL)	9.4 ± 1.6	9.8 ± 1.4	0.035
ΔMPV^a^ (fL)	0.37 (− 0.1–0.89)	0.79 (0.30–1.40)	< 0.001
LVEF (%)	45 ± 9	41 ± 10	< 0.001

Abbreviations: *LVEF* left ventricular ejection fraction, *SCr* serum creatinine at admission, *MPV* mean platelet volume, *ΔMPV* change in mean platelet volume, WBC; white blood cell

^a^Comparison was made using Mann-Whitney *U* test at *P* <  0.05, and these values were described by median with inter-quartile range (25th and 75th percentile)

LVEF was lower in non-survivors than survivors (41 ± 10 vs 45 ± 9, *p* < 0.001).

### Clinical outcomes and ΔMPV

Thirty-day mortality rate was 20% in the non-survivors (Table 1). The frequencies of TVR, stroke, and MI were comparable between groups. Non-survivors had a higher incidence of HF admission compared with survivors (14% vs 2%, *p* < 0.001).

ΔMPV (HR: 1.301 [1.070–1.582], *p* = 0.008), Killip class ≥2, LVEF, history of stroke/TIA and age were independent predictors of long-term mortality in multivariate analysis (Table 3).

**Table 3:** Univariate and multivariate cox proportional hazards analysis for all-cause mortality

Variables	Univariate		Multivariate	
	HR (95% CI)	*P*-value	HR (95% CI)	*P*-value
Age (year)	1.062 (1.045–1.080)	< 0.001	1.049 (1.030.0010–1.069)	< 0.001
Gender (Male)	0.581 (0.391–0.864)	0.007		
History of stroke/TIA	4.263 (2.149–8.457)	< 0.001	2.398 (1.148–5.009)	0.020
History of DM	1.690 (1.138–2.510)	0.009		
History of CAD	1.695 (1.162–2.473)	0.053		
History of HT	1.538 (0.995–2.377)	0.006		
IRA	0.987 (0.823–1.183)	0.888		
Multi-vessel disease	2.001 (1.372–2.915)	< 0.001		
Killip ≥2	3.619 (2.228–5.180)	< 0.001	2.791 (1.597–4.876)	< 0.001
LVEF (%)	0.957 (0.938–0.977)	< 0.001	0.966 (0.945–0.989)	0.003
Hemoglobin (g/dl)	0.832 (0.763–0.907)	< 0.001		
ΔMPV (fL)	1.428 (1.210–1.685)	< 0.001	1.301 (1.070–1.582)	0.008
Serum creatinine (mg/dl)	1.235 (1.091–1.397)	0.001		
Statin usage at discharge	0.413 (0.275–0.619)	< 0.001		
Beta-blocker usage at discharge	0.496 (0.326–0.756)	0.001		
ACE/ARB usage at discharge	0.239 (0.118–0.484)	< 0.001		

Abbreviations: *HR* hazard ratio, *CI* confidence interval, *TIA* transient ischemic attack, *DM* diabetes mellitus, *LVEF* Left ventricular ejection fraction, *HT* hypertension, *CAD* coronary artery disease, *ΔMPV*, change in mean platelet volüme, *ACE* angiotensin-converting enzyme, *ARB* angiotensin receptor blockers, *IRA*: infarct related artery

**Fig. 1:**
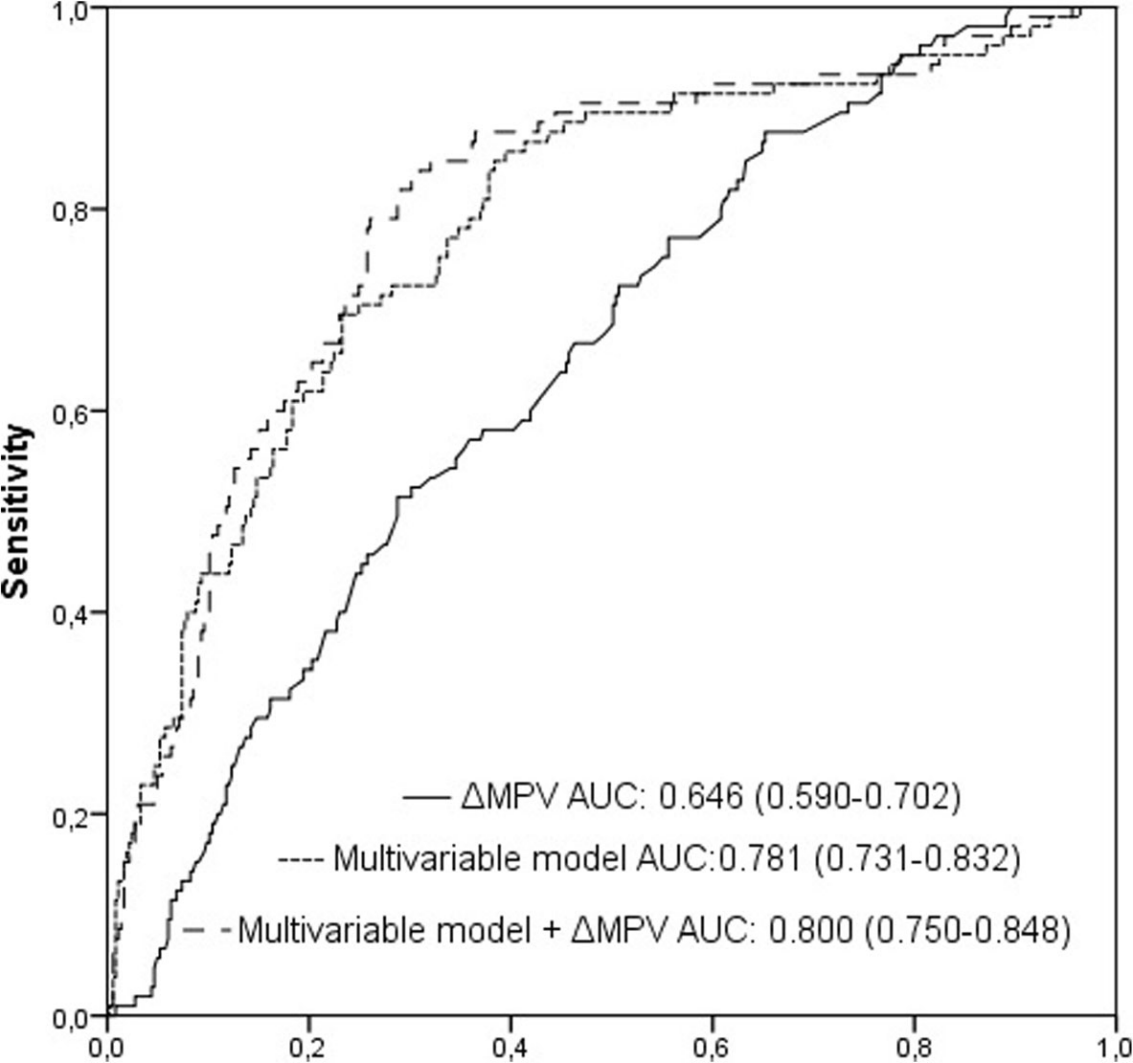
Receiver operating characteristic (ROC) curves for the ΔMPV, multivariable model, and multivariable model plus ΔMPV for predicting all-cause total mortality

**Fig. 2:**
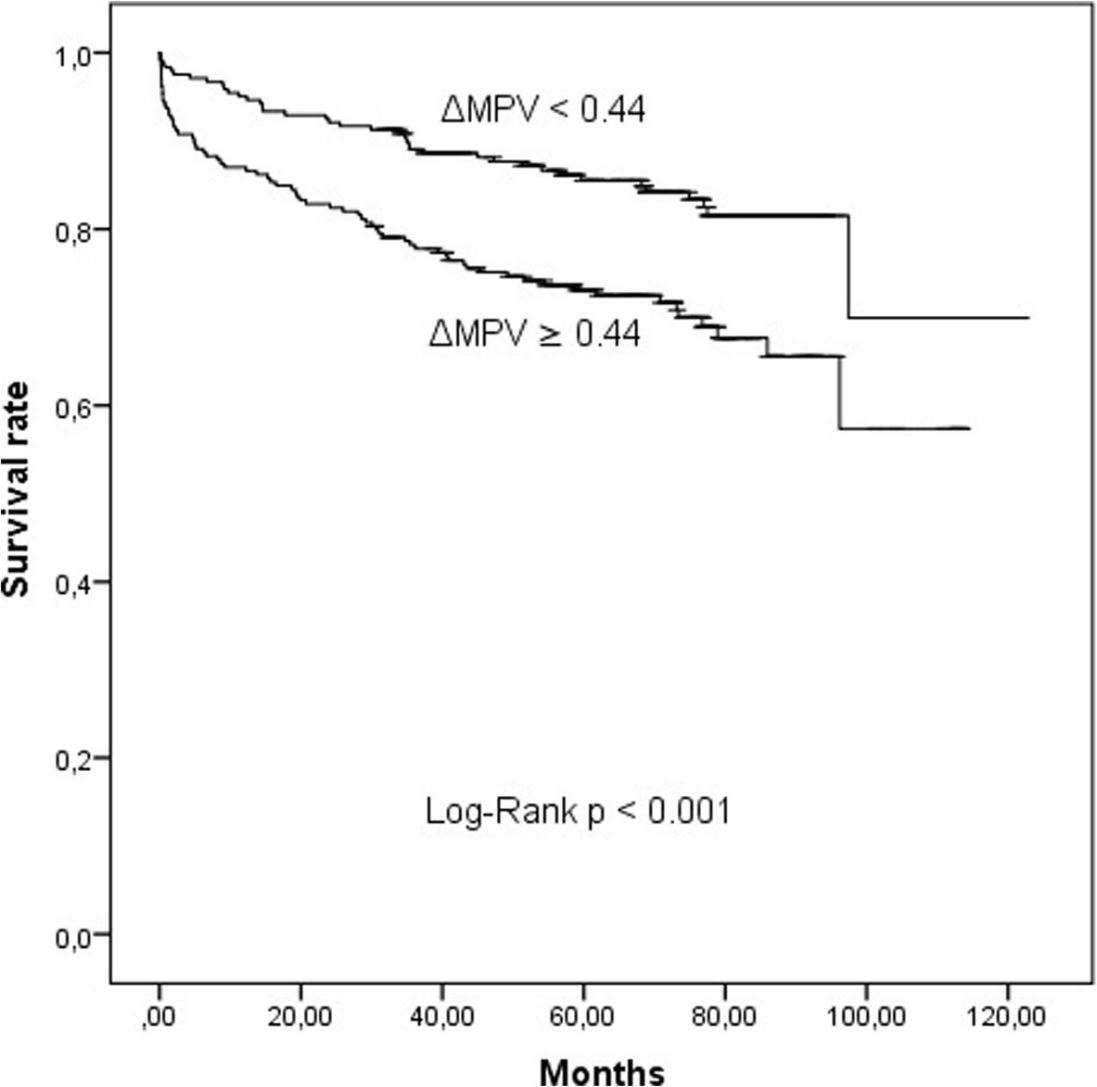
Kaplan-Meier survival curves of all-cause mortality according to ΔMPV

The ROC curve analysis of ΔMPV revealed an area under the curve (AUC) of 0.646 for the prediction of long-term mortality. (Fig. 1). Morever, for long-term mortality, the AUC of a multivariable model that included age, LVEF, Killip class, and history of stroke/TIA was 0.781 (95% CI:0.731–0.832, *p* <  0.001). When ΔMPV was added to a multivariable model, the AUC was 0.800 (95% CI: 0.750–0.848, z = 2.256, difference *p* = 0.0241, Fig. 1). Also, the addition of ΔMPV to a multivariable model was associated with a significant net reclassification improvement estimated at 24.5% (*p* = 0.027) and an integrated discrimination improvement of 0.014 (*p* = 0.0198). Kaplan-Meier survival curves according to a cut-point of ΔMPV (≥ 0.44 fL and < 0.44 fL) are shown in Fig. 2. In the subgroup analysis which was carried out according to the this cut-point, 30-day mortality (2% vs 6%, *p* = 0.009), long-term mortality (16% vs 30%, *p* < 0.001), and heart failure admission (3% vs 7%, *p* = 0.028) were higher in high ΔMPV group compared with low ΔMPV group. There was no significant difference between groups in terms of myocardial reinfarction (8% vs 8%, *p* = 0.756), stroke (3% vs 3%, *p* = 0.998) and TVR (12% vs 12%, *p* = 0.974).

## Discussion

To the best of our knowledge, this is the first study to investigate the association of an in-hospital increase in MPV and long-term mortality in patients with STEMI who were treated with pPCI. In present study, we found that an increase in hospital in MPV at 48–72 h was associated with long-term mortality in these patients.

An increase in MPV at 72 h has been shown Grabovac et al. in STEMI patients [16]. The presence of large PLTs are an indicator of the increased platelet activation. These PLTs are functionally predominantly hyperactivated and have a high granule content including intracellular thromboxane A2, procoagulant surface proteins such as P-selectin and GPIIIa, which is an indicator of prothrombotic state. Also, aggregation in response to collagen or ADP, thromboxane release and membrane expression of P-selectin or GP1b, GPIIb/IIIa have increased in these PLTs [17–19]. There is a relationship between MPV and both proinflammatory and prothrombotic conditions where thrombopoietin and various inflammatory cytokines, such as interleukin (IL) 1, IL-3, and IL-6 and tumor necrosis factor (TNF) a, organize thrombosis. Furthermore, MPV has been shown to be a marker of inflammation in active inflammatory disease [20, 21]. The contribution of PLT to inflammation, in which they perform this by binding to and activating to monocyte, was demonstrated in patients with MI [22, 23].

The mechanism for the enlarged circulating platelets at time after admission remains unclear. Newly generated PLTs arised from bone morrow megakaryocyte are usually bigger as size [18]. The time required for differentiation and maturation of megakaryocyte is about 4–5 days [18, 19]. A period of 24 h is needed for de novo PLTs production and release of these from mature megakaryocytes. Therefore, it is less possible that early increase in MPV after MI is only the result of newly generated bigger PLTs from bone morrow. The human spleen serves as a reservoir for the circulating platelets (about one-third of body PLTs). Morever, MPV in PLTs from human spleen is approximally 20% greater than MPV in circulating PLTs. Thus, the spleen could be reservoir of large PLTs, and may be responsible for fast changes in number of circulating large PLTs under stress settings including intense exercise and stimulation by cytokines or catecholamines [24–27].

In a previous study, it has been shown that spleen thrombocyte release as a fast-acting mechanism increased in circulating MPV shortly after MI [28]. Spleen releasing monocytes and platelets to boost inflammation, has a key role in systemic response ischemic injury, and the another player of cardiosplenic axis is splenic PLTs [28]. In that study, they found that splenectomy broke these changes after MI in mice [28]. It is not clear what is responsible for the initiating of splenic PLTs release and activating of these after MI. The possible responsible mechanisms may be catecholamines and angiotensin II. Swirski et al. showed that these parameters increased in patients with acute MI [29]. Therefore, the elevated levels of angiotensin-II and catecholamin in acute MI setting may lead to splenic PLTs release.

PLTs have been shown to have an important role in inflammation [30]. The inflammatory properties of platelets are mediated by the interaction with large leukocytes [28]. The increased MPV as a reflection of both inflammation and pro-coagulant activity is associated with the risk of stent thrombosis, no-reflow via microthrombi or microvascular damage after PCI, myocardial reinfarction, large infarct size, post-MI heart failure, short and long term mortality in patients with acute MI [31–34].

Kiriş et al. showed there was a relationship between an increase in MPV at 24 h after admission and mortality in NSTEMI patients [11]. In that study, high ΔMPV (> 0.62 fL) patients had higher rate of both 30-day and long-term mortality as found in presented study. The difference between cut-points may be due to different clinical settings (i.e, STEMI vs NSTEMI) and applied treatment strategies. Another study published by Wang et al. demonstrated that serial changes in MPV was related to higher Killip class and no-reflow phenomenon after PCI in patients with STEMI treated with pPCI [35]. MPV at baseline, 30, 60, 90 days, and at 1, 2 and 3 years after PCI was evaluted in unselected coronary artery disease patients [36]. In that study, an increase in MPV over time was associated with long term mortality. In contrast to the their study, we investigated the association of in-hospital increase in MPV with mortality in STEMI patients who were undergoing pPCI. We found that an increase in-hospital MPV after admission was associated with mortality in these patients.

Medications including ACE-I/ARB, beta blockers, statins, and antiplatelet drugs may influence MPV [37, 38]. With regard to ΔMPV, we did not find any difference between patients receiving these drugs and those who did not. Morever, tirofiban usage had no effect on ΔMPV in our study (data not shown). Further research is required to determine the impact of these treatments on ΔMPV.

A high ΔMPV may be indicative of more thrombogenic and active platelets. Also, the presence of it may be a reflection of the increased thrombosis and inflammation. Thus, an increased PLTs size further contribute to the formation of thrombus. Morever, large size PLTs may lead to vasoconstriction and endotelial dysfunction. Therefore, the abovementioned associations may be possible underlying mechanisms of mortality in STEMI patients who were undergoing pPCI.

The present study has a few limitations. This is a retrospective study with a relatively small size, which precludes determining a definitive relationship between ΔMPV and outcomes. The effect of different oral antiplatelts loading dose on MPV was not evaluated in the present study. Also, we could not investigate previous use of nonsteroidal anti-inflammatory drugs before PCI. The DM patients treated with incretin had a significantly lower rate of major cardiovascular events compared to those were not treated by this treatment [39, 40]. As data regarding incretin usage was not present in many patients, its effect on mortality in present study could not be assesed. Moreover, we did not evaluated effect this agents on molecules involved in atherosclerotic plaque stability. Finally, possible selection bias may have impacted these results.

## Conclusion

Rising MPV during hospitalization was associated with long-term mortality in STEMI patients treated with pPCI. We suggest that repeated MPV determinations throughout hospitalization may improve risk stratification in these patients.

## Abbreviations

ACS: Acute coronary syndrome
CAD: Coronary artery diseases
DM: Diabetes mellitus
HF: Heart failure
HT: Hypertension
IDI: Integrated discrimination improvement
LBBB: Leftbundle branch block
LVEF: Left ventricular ejection fraction
MPV: Mean platelet volume
NRI: Net reclassification improvement
PLT: Platelets
pPCI: Primary percutaneous coronary intervention
ROC: Receivers operating characteristic
sCr: Serum creatinine
STEMI: Stelevation myocardial infarction
TİA: Transient ischemic attack
TVR: Target vessel revascularization
WBC: White blood cell
ΔMPV: Change in mean platelet volume
